# Posttreatment Strategy Against Hypoxia and Ischemia Based on Selective Targeting of Nonnuclear Estrogen Receptors with PaPE-1

**DOI:** 10.1007/s12640-021-00441-y

**Published:** 2021-11-19

**Authors:** A. Wnuk, K. Przepiórska, B. A. Pietrzak, M. Kajta

**Affiliations:** Maj Institute of Pharmacology, Polish Academy of Sciences, Department of Pharmacology, Laboratory of Neuropharmacology and Epigenetics, Smętna Street 12, 31–343, Krakow, Poland

**Keywords:** Asphyxia, Stroke, Hypoxia, Ischemia, Membrane estrogen receptors, Apoptosis, Neuroprotection

## Abstract

Newly synthesized Pathway Preferential Estrogen-1 (PaPE-1) selectively activates membrane estrogen receptors (mERs), namely, mERα and mERβ, and has been shown to evoke neuroprotection; however, its effectiveness in protecting brain tissue against hypoxia and ischemia has not been verified in a posttreatment paradigm. This is the first study showing that a 6-h delayed posttreatment with PaPE-1 inhibited hypoxia/ischemia-induced neuronal death, as indicated by neutral red uptake in mouse primary cell cultures in vitro. The effect was accompanied by substantial decreases in neurotoxicity and neurodegeneration in terms of LDH release and Fluoro-Jade C staining of damaged cells, respectively. The mechanisms of the neuroprotective action of PaPE-1 also involved apoptosis inhibition demonstrated by normalization of both mitochondrial membrane potential and expression levels of apoptosis-related genes and proteins such as *Fas*, *Fasl*, *Bcl2*, FAS, FASL, BCL2, BAX, and GSK3β. Furthermore, PaPE-1-evoked neuroprotection was mediated through a reduction in ROS formation and restoration of cellular metabolic activity that had become dysregulated due to hypoxia and ischemia. These data provide evidence that targeting membrane non-GPER estrogen receptors with PaPE-1 is an effective therapy that protects brain neurons from hypoxic/ischemic damage, even when applied with a 6-h delay from injury onset.

## Introduction

Acute stroke is a commonly used name for a cerebrovascular accident. Cerebrovascular accidents are the 2^nd^ leading cause of death and a leading cause of disability worldwide. Research has been shown that up to 15 million people suffer from stroke annually, that more than 5 million of whom die, and that another 5 million of whom remain disabled for the rest of their lives (World Health Organisation (n.d.), Stroke, cerebrovascular accident). Approximately three-quarters of all strokes occur in persons aged ≥ 65 years, but strokes can occur at any age (Yousufuddin and Young 2019). The 3 main types of stroke are ischemic stroke, hemorrhagic stroke, and transient ischemic attack. Ischemic stroke accounts for 87% of all stroke cases and is caused by the blood clot resulting in the loss of blood circulation (Centers for Disease Control and Prevention. Types of stroke 2020). The gold standard for stroke treatment is an injection of recombinant tissue plasminogen activator (rtPA), which is also called alteplase. rtPA can be given up to 4.5 h after stroke symptoms start and has a long list of contraindications, such as intracranial hemorrhage and cerebral edema (Jilani and Siddiqui 2021). Consequently, only 5% of stroke patients can be treated with rtPA (Miller et al. 2011; Frendl and Csiba 2011).

Perinatal asphyxia is a condition characterized by fetal oxygen deprivation that leads to the death of approximately 1 million children and disability of another 1 million annually (Manandhar and Basnet 2019). It occurs during the antepartum, intrapartum, or perinatal period. First, a reduced oxygen supply (hypoxia) impairs neuronal cell function. Next, prolonged hypoxia leads to myocardial cell dysfunction, and cardiac failure causes ischemia in peripheral organs and the brain (Rainaldi and Perlman 2016). The current therapy for perinatal asphyxia must occur within 6 h and is based on therapeutic hypothermia (selective head or systemic cooling) and oxygen therapy (Zubcevic et al. 2015). Recent studies have shown that therapeutic hypothermia may increase the risk of persistent pulmonary hypertension in neonates (Vijverberg Joanna et al. 2021).

Several pathways are involved solely or in combination in hypoxia/ischemia injury, including apoptosis, necrosis, and oxidative stress. During the lack of blood supply due to hypoxia or ischemia, in addition to acute necrosis, apoptosis appears to determine the fate of cells (Wu et al. 2018). Apoptosis contributes to a significant proportion of neuronal death in the ischemic penumbra during blood flow restoration, which is also called reperfusion. Suppression of glucose and oxygen delivery causes activation of both prosurvival and proapoptotic signaling cascades (Chen et al. 2009). Moreover, reactive oxygen species (ROS) are strongly associated with brain injury after hypoxia and ischemia. Immediately after acute oxygen and/or glucose deprivation, ROS production rapidly increases as a consequence of an imbalance between pro-oxidants and antioxidants and overwhelmed antioxidant defenses. ROS can damage cell structures, including DNA, proteins, lipids, and membranes leading to apoptosis and necrosis. Moreover, during reperfusion, brain cell oxygenation increases and leads to a second burst of ROS generation (Li and Yang 2016).

Sex dimorphism seems to be an important factor in ischemic stroke outcomes. Women (45–74 years old) have a lower risk of stroke incidence and lower mortality than men of the same age (Jiang et al. 2020). Many studies have confirmed that estrogen receptors (ERs) have a neuroprotective capacity against ischemia reperfusion. However, therapy based on estrogen itself or on SERMs (selective ER modulators), e.g., tamoxifen and raloxifene, has several side effects (e.g., venous thromboembolic events and an increased risk of breast/uterine ER-dependent cancer). Sex dimorphism is caused by activation of the nuclear ERs responsible for regulating transcriptional processes and the expression of several genes in peripheral tissues in response to hormonal effects. In addition to the nuclear localization of classical nuclear ERs, receptors are also localized on cell membranes, i.e., mERα, mERβ, ER-X, GPER1, and GqmER, mainly in the nervous, metabolic, and cardiovascular systems. Stimulation of mERs has been shown to activate various neuronal survival-related rapid signaling pathways and prevent neuronal cell death through the kinases PI3K and ERK/MAPK and the tyrosine cascade as well as membrane-associated molecules such as ion channels and G-protein-coupled receptors (De Butte-Smith et al. 2012; Saczko et al. 2017). This action is called nonnuclear or membrane activity. In recent years, these differences have rendered membrane ERs ideal pharmacological targets devoid of the negative side effects associated with nuclear activity.

In 2016, Madak-Erdogan et al. designed Pathway Preferential Estrogen-1 (PaPE-1), which provides beneficial metabolic and vascular effects without stimulating reproductive tissues. PaPE-1 ((S)-5-(4-hydroxy-3,5-dimethyl-phenyl)-indan-1-ol) preferably interacts with the mER signaling pathway (50,000 times less bound to nuclear receptors); specifically, PaPE-1 selectively interacts with mERα and mERβ without activating the nuclear signaling pathway of the controversial GPER1 receptor. Another important feature of PaPE-1 is the short lifetime of ER-PaPE-1 complexes; the half-life of the ER-E2 (17β-estradiol) complex is nearly 30 h, whereas that of the ER-PaPE-1 complex is less than 1 min. This short time is sufficient to activate kinase cascades and at the same time is insufficient to promote transcription *via* nuclear receptors. Therefore, PaPE-1 does not induce ERα or ERK2 recruitment to gene enhancers or stimulate the expression of proliferation-associated genes, as observed with E2. However, similar to E2, PaPE-1 strongly activates the MAPK and mTOR pathways. The neuroprotective capacity of PaPE-1 against Amyloid β (Aβ)-induced neurotoxicity and apoptosis has already been evidenced by our group (Wnuk et al. 2020a). We showed that following 24 h of exposure, PaPE-1 inhibited Aβ-evoked effects, as shown by reduced parameters of neurotoxicity, oxidative stress, and apoptosis. Moreover, PaPE-1 normalized the Aβ-induced loss of mitochondrial membrane potential and restored the BAX/BCL2 ratio, suggesting that the anti-Alzheimer’s disease (AD) capacity of PaPE-1 particularly relies on inhibition of the mitochondrial apoptotic pathway.

Since a posttreatment paradigm is the most relevant approach to improve hypoxia-/ischemia-based injuries, the present study aims to identify the neuroprotective potential and mechanisms of action of PaPE-1 as a posttreatment therapy in cellular models of ischemic stroke and perinatal asphyxia.

## Materials and Methods

### Materials

Phosphate-buffered saline (PBS) was purchased from Biomed Lublin (Lublin, Poland). B27 and neurobasal media were obtained from Gibco (Grand Island, NY, USA). The ROS-Glo™ H_2_O_2_ assay was obtained from Promega (Madison, WI, USA). The cytotoxicity detection kit and neutral red dye were purchased from Roche Diagnostics GmbH (Mannheim, Germany). ELISA kits for FAS, FASL, BAX, BCL2, and GSK3B were purchased from Bioassay Technology Laboratory (Shanghai, China). Culture plates were obtained from TPP Techno Plastic Products AG (Trasadingen, Switzerland). L-Glutamine, fetal bovine serum (FBS), dimethyl sulfoxide (DMSO), 4-(2-hydroxyethyl)-1-piperazineethanesulfonic acid (HEPES), 3-[(3-cholamidopropyl)dimethylammonio]-1-propanesulfonate hydrate (CHAPS), ammonium persulfate, N,N,N′,N′-tetramethylethane-1,2-diamine (TEMED), 2-amino-2-(hydroxymethyl)-1,3-propanediol (Trizma base), DL-dithiothreitol, sodium deoxycholate, protease inhibitor (ethylenediaminetetraacetic acid-free), bromophenol blue, radioimmunoprecipitation assay buffer (RIPA) buffer, Fluoro-Jade C, PaPE-1 ((S)-5-(4-hydroxy-3,5-dimethyl-phenyl)-indan-1-ol, 0.01–10 μM), thiazolyl blue tetrazolium bromide, protease inhibitor cocktail for mammalian tissues, and polyornithine were obtained from Sigma-Aldrich (St. Louis, MO, USA). JC-10 was purchased from Abcam (Cambridge, UK). The RNeasy Mini Kit was obtained from Qiagen (Hilden, Germany). The RNA quality (integrity) was analyzed using PrimePCR™ RNA Quality Probe Assay, Mouse (Bio-Rad, USA). A High-Capacity cDNA-Reverse Transcription Kit, TaqMan Gene Expression Master Mix, and TaqMan probes for specific genes encoding *Hprt*, *β-actin, Gapdh,* *Fas*, *Fasl*, *Bax*, *Bcl2*, and *Gsk3b* were obtained from Thermo Fisher Scientific (Waltham, MA, USA).

## Methods

### Primary Neuronal Cell Culture

Primary neocortical cultures were prepared from CD-1^®^ IGS Swiss mouse embryos at 15 days of gestation (Charles River, Germany) as previously described (Kajta et al. 2006; Wnuk et al. 2020a, b). The cortices were mechanically and then enzymatically fragmented with 0.1% trypsin for 15 min at 37 °C. Next, the cells were centrifuged for 5 min at 1,500 × g and plated in medium containing 10% fetal bovine serum. The cells were cultured on plates (TPP Techno Plastic Products AG, Switzerland) covered by poly-*L*-ornithine in neurobasal medium (Thermo Fisher Scientific, USA) with *L*-glutamine (Sigma–Aldrich, USA), B27 (Thermo Fisher Scientific, USA), and penicillin–streptomycin antibiotics (Sigma-Aldrich, USA) at 37 °C in a humidified atmosphere containing 5% CO_2_ for 7 days in vitro (DIV). The density of the cells was 2.0 × 10^5^ cells per cm^2^. All animals used in the research were maintained according to the principles of the Three Rs in compliance with European Union Legislation (Directive 2010/63/EU, amended by Regulation (EU) 2019/1010).

### Experimental Models of Hypoxia and Ischemia

To induce hypoxic conditions, the cell medium was replaced with standard medium, whereas to induce ischemia, the cell medium was replaced with medium without glucose. Then, the procedure was the same for both experimental models. The cells were placed in a prewarmed and humidified hypoxia modular incubator chamber (Billups-Rothenberg, Inc., CA, USA) with 95% N_2_/5% CO_2_ for 6 h. The O_2_ level was measured with an oxygen analyzer (Greisinger, Germany) and reached less than 0.5%. After 6 h of hypoxic/ischemic conditions, i.e., at the reoxygenation period, the culture medium was replaced immediately with standard medium for 18 h (Wnuk, Przepiórska et al. 2021).

### Treatment

Cell cultures were treated with PaPE-1 ((S)-5-(4-hydroxy-3,5-dimethyl-phenyl)-indan-1-ol) at a concentration of 0.01–10 μM. During reoxygenation, cells were cultured in a humidified incubator (New Brunswick Scientific, NJ, USA). PaPE-1 was dissolved in DMSO, not exceeding a concentration of 0.1% in the culture medium, as previously described (Wnuk et al. 2020a).

### Assessment of Lactate Dehydrogenase Release

The cytotoxicity in neuronal cell cultures was assessed by measuring lactate dehydrogenase (LDH) release into the cell culture medium as previously described (Kajta et al. 2019). After experiments, the supernatant was collected and incubated with the relevant reaction mixture for 60 min at room temperature. The absorbance was measured after 30 and 60 min at 490 nm with the use of an Infinite M200 PRO microplate reader (Tecan Mannedorf, Switzerland), and the results were analyzed by i-control software. Data were normalized to the vehicle-treated cells (DMSO 0.1%) and presented as a percentage of the control ± SEM. The intensity of the red color was proportional to LDH release from damaged cells.

### Caspase-3 Activity Measurement

Caspase-3 activity was measured in neocortical cell cultures 18 h after treatment with PaPE-1 (Kajta et al. 2009; Rzemieniec et al. 2019). To initiate cell lysis, caspase assay buffer (containing 50 mM HEPES, pH 7.4, 100 mM NaCl, 0.1% CHAPS, 1 mM EDTA, 10% glycerol, and 10 mM DTT) was added to each well. After this step, cell lysates were incubated with acetyl-Asp-Glu-Val-Asp *p-*nitroanilide (Ac-DEVD-*p*NA) for 60 min at 37 °C. The reaction was based on the hydrolysis of Ac-DEVD-*p*NA by caspase-3 and the release of *p*-nitroanilide detected at 405 nm. Absorbance measurements were performed using an Infinite M200 PRO microplate reader (Tecan, Mannedorf, Switzerland). The results were analyzed by i-control software, normalized to the absorbance of vehicle-treated cells (DMSO 0.1%), and presented as a percentage of the control ± SEM. The amount of the reaction product was proportional to caspase-3 activity.

### Measurement of Neutral Red Uptake

To assess the viability of neuronal cell cultures after hypoxia/ischemia, neutral red dye was used. This dye which is able to bind to the lysosomes of viable cells, as previously described (Szychowski et al. 2019). First, a 10% neutral red solution was prepared, filtered, and heated to 37 °C. Then, the solution was added to the cells and was followed by 2 h of incubation at 37 °C. Then, the neuronal cultures were washed with PBS and incubated with acidified ethanol solution (50% ethanol, 1% acetic acid, 49% H_2_O) for 10 min. The absorbance was measured at a wavelength of 540 nm using an Infinite M200 PRO microplate reader (Tecan, Mannedorf, Switzerland) and i-control software. Data were normalized to vehicle-treated cells and presented as a percentage of the control ± SEM. The extracted dye was proportional to the number of living cells.

### Assessment of MTT Reduction

This colorimetric assay is based on reducing yellow 3-(4,5-dimethylthiazol-2-yl)-2,5-diphenyltetrazolium bromide (MTT) to purple formazan through oxidoreductase enzymes. The purple color intensity of dissolved formazan correlates with the mitochondrial activity of neuronal cells. After the experiment, MTT solution was added to the cell cultures for incubation at 37 °C for 1 h. Next, the cell medium was replaced with DMSO to dissolve formazan crystals. The absorbance was measured at 570 nm with the use of an Infinite M200 PRO microplate reader (Tecan Mannedorf, Switzerland), and the data were analyzed with i-control software. The results were normalized to vehicle-treated cell cultures and presented as the percentage of the control ± SEM.

### Measurement of Degenerating Neurons

To determine the degree of neurodegeneration under hypoxic/ischemic conditions and after PaPE-1 treatment, Fluoro-Jade C (FJ-C) staining was used. This novel method enables in vitro detection of cytotoxicity using fluorochromatic dyes. The solution was prepared by mixing FJ-C with distilled water, and the culture medium was replaced with a mixture. After 1 h of incubation, the fluorescence was measured at Ex/Em = 490/525 by an Infinite M200 PRO microplate reader (Tecan Mannedorf, Switzerland). The results analyzed by i-control software were normalized to data from vehicle-treated cells and presented as the percentage of the control ± SEM.

### Measurement of ROS Formation

The ROS-Glo™ H_2_O_2_ Assay (Promega, Madison, WI, USA) was used to assess the level of reactive oxygen species (ROS) in neocortical cells exposed to hypoxia/ischemia and PaPE-1 treatment. ROS-Glo™ H_2_O_2_ substrate was added to cell cultures at the end of the treatment and reacted with H_2_O_2_ present in our samples. The reaction product, a luciferin precursor, was converted to luciferin by adding ROS-Glo™ Detection Solution. The bioluminescence was measured with the use of a GloMax® Navigator Microplate Luminometer (Promega, Madison, WI, USA), and the light signal was proportional to the amount of H_2_O_2_ in cultured cells. The data were normalized to the bioluminescent signal intensity of vehicle-treated cells and expressed as a percentage of the control ± SEM.

### Assessment of the JC-10 Concentration

JC-10 is a widely used dye that aggregates in mitochondria and changes its color from green to orange, which is detected as a membrane potential increase. To monitor mitochondrial membrane potential changes after hypoxic/ischemic episodes and PaPE-1 treatment, the JC-10 Mitochondrial Membrane Potential Assay Kit (Abcam, Cambridge, UK) was used. JC-10 dye-loading solution was added to each well and incubated with cell cultures for 1 h at 37 °C. After adding Assay Buffer B, the fluorescence was monitored at Ex/Em = 490/525 and Ex/Em = 540/590 nm with an Infinite M200 PRO microplate reader (Tecan Mannedorf, Switzerland). The fluorescence intensity was used for the ratio analysis, and the results are presented as a percentage of the control ± SEM. Data were normalized to the fluorescence intensity of vehicle-treated cells.

### qPCR Analysis of mRNA-Specific for Genes Encoding Apoptosis-Related Factors

To extract total RNA from neocortical cells, a Qiagen RNeasy Mini Kit (Hilden, Germany) was used as previously described (Wnuk et al. 2019). The quantity of RNA was spectrophotometrically determined at 260 nm and 260/280 nm (ND/1000 UV/Vis; Thermo Fisher NanoDrop, Waltham, MA, USA). Two-step qPCR consisting of reverse transcription and qPCR was performed using the CFX96 Real-Time System (Bio-Rad, Hercules, CA, USA). Total RNA was reverse transcribed with a High-Capacity cDNA Reverse Transcription Assay (Thermo Fisher Scientific, Waltham, MA, USA). The collected cDNA was then amplified with TaqMan probes of the specific genes encoding *Hprt*, *β-actin, Gapdh,* *Fas*, *Fasl*, *Bax*, *Bcl2*, and *Gsk3b*. Twenty microliters of the final amplification mixture consisted of 10 µl of FastStart Universal Probe Master Mix (Roche, Basel, Switzerland), 8 µl of RNAse-free water, 1 µl of template cDNA, and 1 µl of the TaqMan probe. The qPCR procedure was performed as follows: 2 min at 50 °C, 10 min at 95 °C followed by 40 cycles of 15 s at 95 °C and 1 min at 60 °C. The obtained data were analyzed according to the delta Ct method. To choose the reference gene, RefFinder was used (Xie et al. 2012), which led to the selection of *Hprt* as a reference gene.

### Enzyme-Linked Immunosorbent Assays for Apoptosis-Related Factors

The expression of apoptosis-related factors, i.e., FAS, FASL, BAX, BCL2, and GSK3β, was assessed by enzyme-linked immunosorbent assay (ELISA) after hypoxia/ischemia and PaPE-1 treatment as previously described (Kajta et al. 2020). Cells were lysed with ice-cold RIPA lysis buffer and protease inhibitor cocktail. Next, cell lysates were sonicated and centrifuged at 15,000 × g for 20 min at 4 °C. The protein concentration in the collected supernatant was measured using Bradford reagent and bovine serum albumin as a standard. Antigens from our samples were attached to the precoated wells with mouse antibody surfaces. Biotin-conjugated polyclonal antibodies specific for FAS, FASL, BAX, BCL2, and GSK3β were added to each well. After every step, the plates were washed to remove any nonspecifically bound proteins and antibodies. Then, streptavidin-HRP attached to biotinylated antibodies and the addition of substrate solution caused a color change from blue to yellow. The absorbance was measured with an Infinite M200 PRO microplate reader (Tecan Mannedorf, Switzerland), and the results were expressed as a percentage of the control value ± SEM and in terms of picograms per milligram of total protein. The intensity of yellow color was proportional to the amount of a specific protein.

### Statistical Analysis of the Data

Statistical tests were performed on raw data. The results are expressed as the mean absorbance, fluorescence intensity (in arbitrary units) and luminescence signal per well containing 50,000 cells for analyses of LDH release, caspase-3 activity, Fluoro-Jade C, ROS activity, neutral red staining, MTT, and mitochondrial membrane potential as fluorescence units per 1.5 million cells for qPCR or as the picograms per miligram of total protein for the ELISAs. One-way analysis of variance (ANOVA) was preceded by Levene’s test of homogeneity of variances and was used to determine overall significance. Differences between the control and experimental groups were assessed with a post hoc Newman–Keuls test. Significant differences were indicated as follows: ^*^p < 0.05, ^**^p < 0.01, ^***^p < 0.001 compared to the normoxic control; ^#^*p* < 0.05, ^##^*p* < 0.01, ^###^*p* < 0.001 compared to the cell cultures exposed to hypoxia; and ^^^*p* < 0.05, ^^^^*p* < 0.01, and ^^^^^*p* < 0.001 compared to the cell cultures exposed to ischemia. The results are expressed as the mean ± SEM of 3 independent experiments. The number of replicates ranged from 6 to 12.

## Results

### PaPE-1 Inhibited Lactate Dehydrogenase (LDH) Release and Caspase-3 Activity in Neocortical Cell Cultures Exposed to Hypoxia/Ischemia

**Fig. 1:**
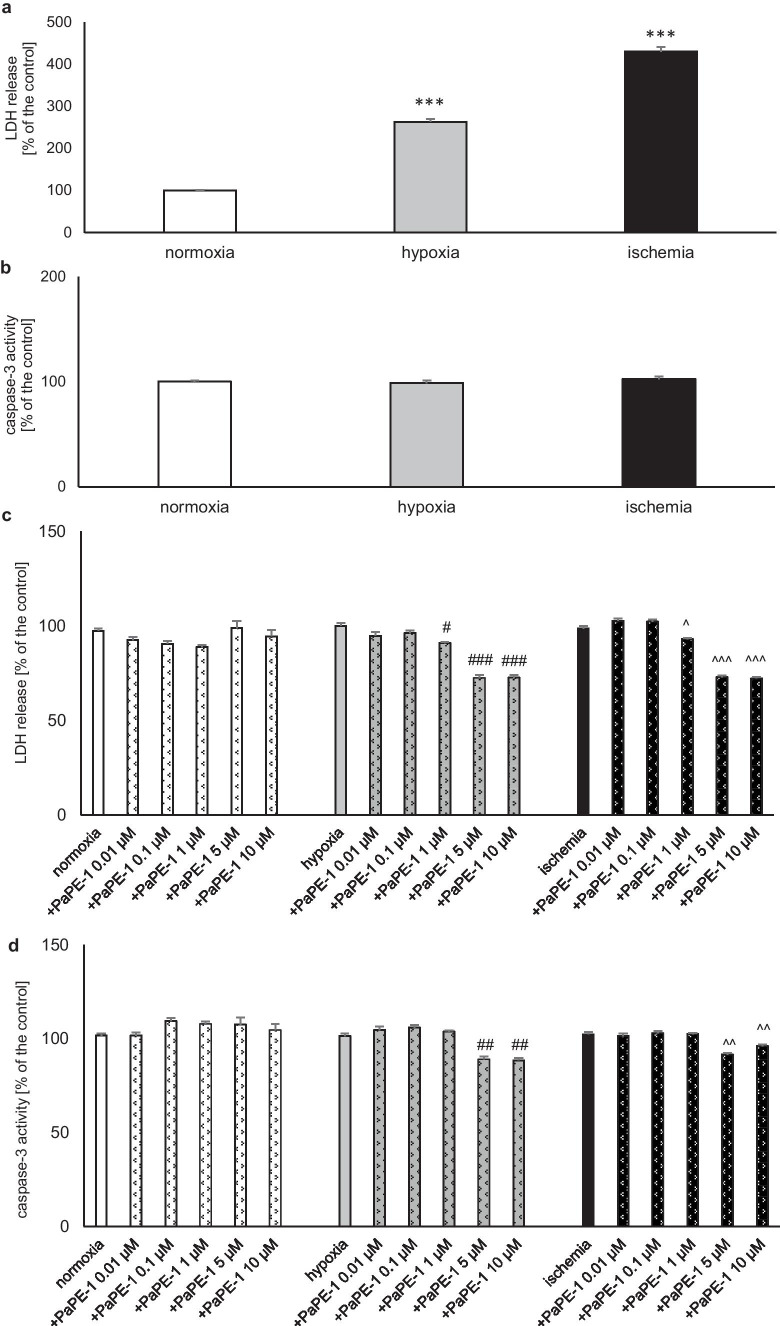
PaPE-1 inhibited LDH release and caspase-3 activity in neocortical cell cultures exposed to hypoxia/ischemia injury. The results are presented as a percentage of the control. Each bar represents the mean ± SEM of 3 independent experiments, with 7 to 10 replicates per group. ^***^p < 0.001 compared to the normoxic control; ^#^*p* < 0.05, ^##^*p* < 0.01, ^###^*p* < 0.001 compared to the cell cultures exposed to hypoxia; ^^^*p* < 0.05, ^^^^*p* < 0.01, ^^^^^*p* < 0.001 compared to the cell cultures exposed to ischemia

In this study, after 6 h of hypoxia/ischemia, 18 h of reoxygenation was applied. Hypoxia and ischemia conditions induced LDH release to 263% and 430% of the normoxic value, respectively (Fig. 1a). Caspase-3 activity remained unchanged under hypoxic/ischemic conditions (Fig. 1b). In this study, PaPE-1 was added during the reoxygenation period as a posttreatment therapy 6 h after the initial injury. Under hypoxic and ischemic conditions, PaPE-1 (1–10 μM) inhibited LDH release to 72–93% of the hypoxia/ischemia value, whereas concentrations of 0.01 μM and 0.1 μM did not change the LDH release level (Fig. 1c). For caspase-3 activity, only 5 and 10 μM PaPE-1 reduced the value to 88–96% of the control value (Fig. 1d). Under normoxic conditions, PaPE-1 used at concentrations ranging from 0.01 to 10 μM did not change LDH release or caspase-3 activity.

Since the most promising concentrations were 5 and 10 µM PaPE-1, these concentrations were used in the next experiments.

### PaPE-1 Partially Reversed Hypoxia/Ischemia-Evoked Neurodegeneration

**Fig. 2:**
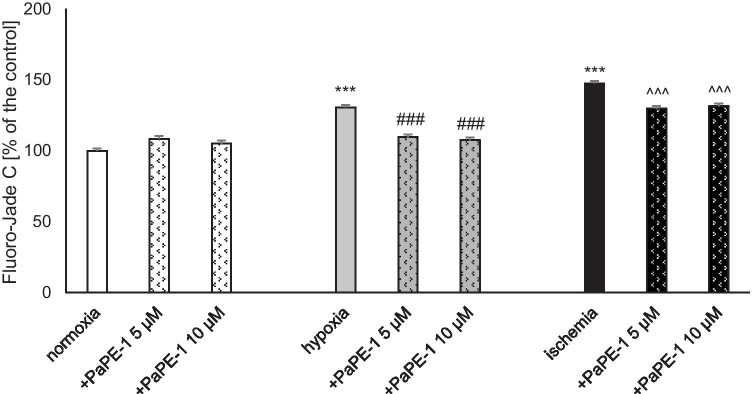
PaPE-1 (5 and 10 μM) partially reversed hypoxia/ischemia-evoked neurodegeneration measured using Fluoro-Jade C staining in 7 DIV neocortical cultures. The results are presented as a percentage of the control. Each bar represents the mean ± SEM of 3 independent experiments, with 8–12 replicates per group. ^***^p < 0.001 compared to the normoxic control; ^###^*p* < 0.001 compared to the cell cultures exposed to hypoxia; ^^^^^*p* < 0.001 compared to the cell cultures exposed to ischemia

The degree of neurodegeneration was measured using Fluoro-Jade C staining. Six hours of hypoxic or ischemic conditions followed by 18 h of reoxygenation elevated the degree of neurodegeneration to 130% and 147% of the normoxic value, respectively. Administration of PaPE-1 at concentrations of 5 and 10 μM reversed neurodegeneration by decreasing the parameter values to 110 and 108%, respectively, under hypoxia and 130 and 132%, respectively, under ischemia. Under normoxic conditions, PaPE-1 at concentrations of 5 and 10 μM did not change the degree of degenerating neurons (Fig. 2).

### PaPE-1 Inhibited Hypoxia/Ischemia-Evoked Loss of Mitochondrial Membrane Potential

**Fig. 3:**
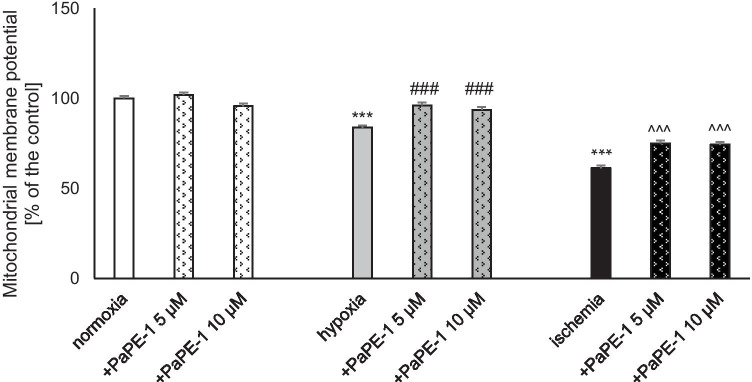
PaPE-1 (5 and 10 μM) inhibited hypoxia/ischemia-evoked loss of mitochondrial membrane potential in 7 DIV neocortical cultures. The results are presented as a percentage of the control. Each bar represents the mean ± SEM of 3 independent experiments, with 10–12 replicates per group. ^***^p < 0.001 compared to the normoxic control; ^###^*p* < 0.001 compared to the cell cultures exposed to hypoxia; ^^^^^*p* < 0.001 compared to the cell cultures exposed to ischemia

JC-10 labeling showed that hypoxia and ischemia resulted in a decrease in mitochondrial membrane potential, reaching 84 and 61% of the control value, respectively. Under hypoxic conditions, PaPE-1 administration (5 and 10 μM) normalized the mitochondrial membrane potential to 94–96% of the control value. In the ischemic model, PaPE-1 (5 and 10 μM) partially inhibited ischemia-evoked loss of mitochondrial membrane potential to 75% of the normoxic value. During normoxia, PaPE-1 at concentrations of 5 μM and 10 μM did not change this parameter (Fig. 3).

### PaPE-1 Partially Reversed the Hypoxia/Ischemia-Evoked Decrease in Cell Metabolic Activity

**Fig. 4:**
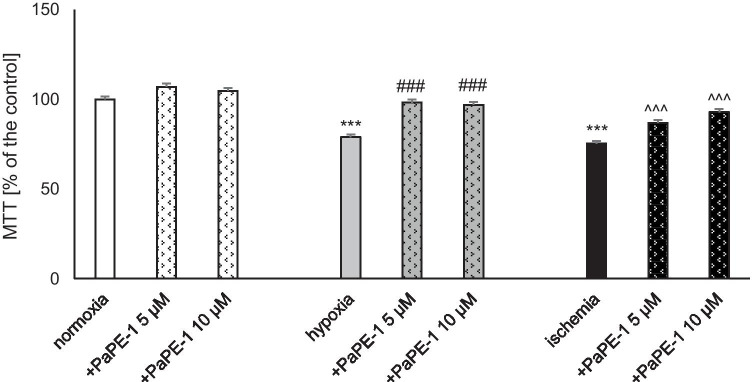
PaPE-1 (5 and 10 μM) partially reversed the hypoxia/ischemia-evoked decrease in cell metabolic activity measured using MTT staining in 7 DIV neocortical cultures. The results are presented as a percentage of the control. Each bar represents the mean ± SEM of 3 independent experiments, with 10–12 replicates per group. ^***^p < 0.001 compared to the normoxic control; ^###^*p* < 0.001 compared to the cell cultures exposed to hypoxia; ^^^^^*p* < 0.001 compared to the cell cultures exposed to ischemia

MTT staining revealed that hypoxia and ischemia led to a loss of cell metabolic activity. As a consequence of hypoxia, a decrease to 79% of the normoxic value was observed. Comparably, ischemia resulted in 75% of the normoxic cells’ metabolic activity. PaPE-1 (5 and 10 μM) applied for the reoxygenation period increased the metabolic activity of cells subjected to hypoxia and ischemia. When administered after hypoxic insult, the compound normalized the tested parameter to control values, i.e., 96–98% of the control. Under ischemic conditions, 5 μM PaPE-1 increased cell metabolic activity to 87%, and 10 μM PaPE-1 improved it to 92% of the normoxic value. Under normoxic conditions, PaPE-1 did not induce changes in the MTT parameter (Fig. 4).

### PaPE-1 Inhibited Hypoxia-/Ischemia-Evoked Cell Viability Decrease

**Fig. 5:**
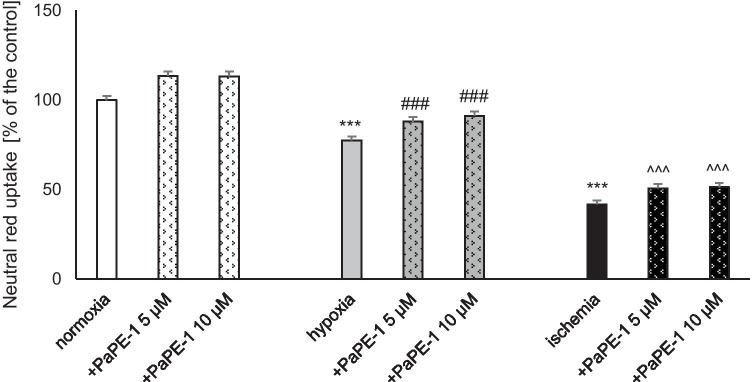
PaPE-1 (5 and 10 μM) inhibited the hypoxia-/ischemia-evoked decrease in cell viability measured using neutral red uptake in neocortical cultures at 7 DIV. The results are presented as a percentage of the control. Each bar represents the mean ± SEM of 3 independent experiments, with 12 replicates per group. ^***^p < 0.001 compared to the normoxic control; ^###^*p* < 0.001 compared to the cell cultures exposed to hypoxia; ^^^^^*p* < 0.001 compared to the cell cultures exposed to ischemia

In this study, neutral red staining revealed that 6 h of hypoxia/ischemia followed by 18 h of reoxygenation decreased cell viability to 78 and 41% of the normoxic value, respectively. PaPE-1 applied at the beginning of reoxygenation improved the viability of neurons subjected to both hypoxic and ischemic conditions. In the hypoxia model, a 5-μM concentration of the tested compound increased the measured parameter to 88% (10% increase), while treatment with a 10-μM concentration resulted in a rise to 91% (13% increase). PaPE-1 also protected cortical neurons after ischemic episodes by increasing cell viability to 51% (10% increase) after treatment with both 5 and 10 μM PaPE-1. Under normoxic conditions, neither 5 nor 10 μM PaPE-1 changed cell viability (Fig. 5).

### PaPE-1 Partially Reversed Hypoxia/Ischemia-Evoked ROS Formation

**Fig. 6:**
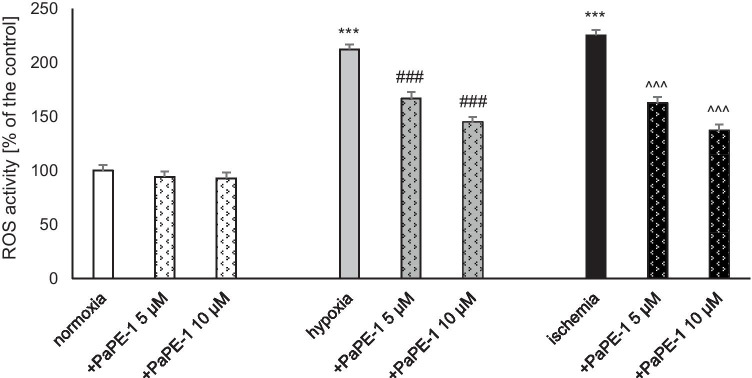
PaPE-1 (5 and 10 μM) partially reversed hypoxia/ischemia-evoked ROS formation in neocortical cultures at 7 DIV. The results are presented as a percentage of the control. Each bar represents the mean ± SEM of 3 independent experiments, with 10–12 replicates per group. ^***^p < 0.001 compared to the normoxic control; ^###^*p* < 0.001 compared to the cell cultures exposed to hypoxia; ^^^^^*p* < 0.001 compared to the cell cultures exposed to ischemia

In neocortical cell cultures subjected to hypoxia or ischemia, increased ROS formation was observed. In both models, ROS formation doubled in response to oxygen or oxygen and glucose deprivation (212% of the normoxic value under hypoxia and 225% of the normoxic value under ischemia). Under hypoxia, treatment with PaPE-1 at concentrations of 5 and 10 μM substantially reduced the parameter to 145–167%. Similarly, under ischemic conditions, PaPE-1 also lowered the ROS formation, resulting in a decrease to 137–162%. Moreover, under normoxic conditions, neither concentration of PaPE-1 altered ROS activity in neocortical cells (Fig. 6).

### PaPE-1 Normalized the Hypoxia/Ischemia-Dysregulated Expression Levels of Apoptosis-Related Genes and Proteins

**Fig. 7:**
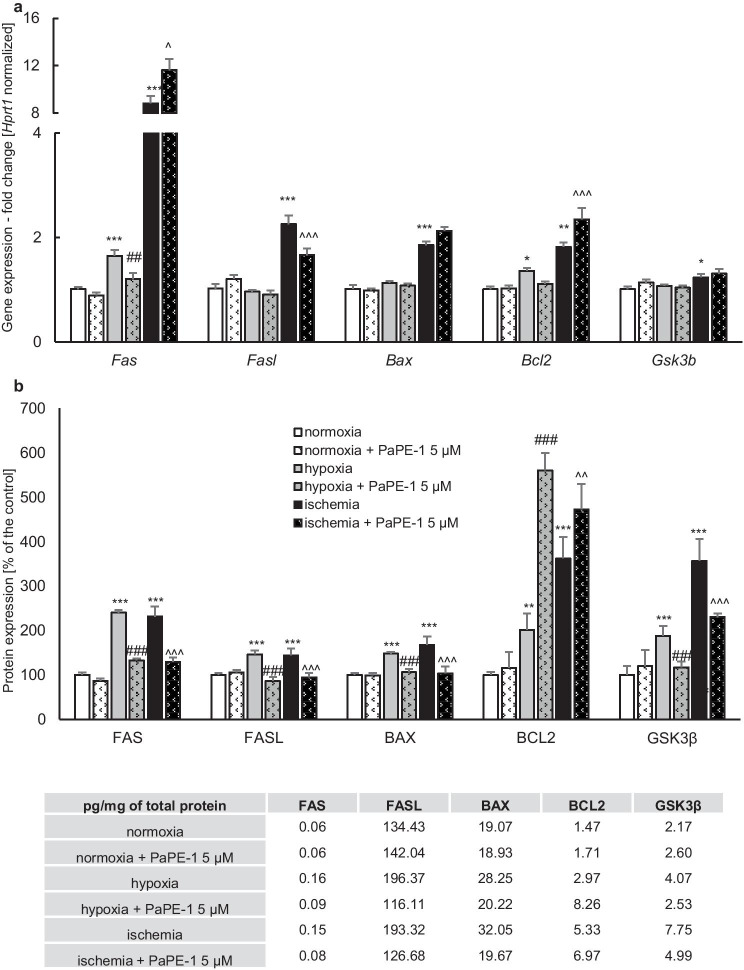
PaPE-1 (5 μM) affected the hypoxia/ischemia-induced increased expression levels of apoptosis-related genes and proteins in mouse neocortical cells at 7 DIV. Receptor levels were measured using qPCR (**a**) and specific ELISAs (**b**). Each result is presented as a fold change, a percentage of the control or in terms of pg per mg of total protein. Each bar represents the mean ± SEM of 3 independent experiments, with 6 replicates per group. ^*^p < 0.05, ^**^p < 0.01, ^***^p < 0.001 compared to the normoxic control; ^##^*p* < 0.01, ^###^*p* < 0.001 compared to the cell cultures exposed to hypoxia; ^^^*p* < 0.05, ^^^^^*p* < 0.001 compared to the cell cultures exposed to ischemia

In our study, exposure to hypoxia enhanced the mRNA expression levels of apoptosis-related genes, i.e., *Fas* (0.64-fold increase) and *Bcl2* (0.36-fold increase), although the expression of *Fasl*, *Bax*, and *Gsk3b* remained unchanged. Treatment with PaPE-1 (5 μM) decreased only the expression of *Fas* to 1.2-fold. In ischemia-exposed cells, the expression of apoptosis-related genes substantially increased, i.e., *Fas* (7.82-fold increase), *Fasl* (1.25-fold increase), *Bax* (0.85-fold increase), *Bcl2* (0.81-fold increase), and *Gsk3b* (0.23-fold increase). After PaPE-1 (5 μM) administration, the expression of *Fasl* decreased to 1.66-fold, and the expression of *Fas* and *Bcl2* increased to 11.6-fold and 2.35-fold, respectively. PaPE-1 did not change the mRNA expression levels of *Bax* and *Gsk3b*. In normoxic cells treated with PaPE-1, the expression of all tested apoptosis-related genes did not change (Fig. 7a).

ELISA analyses showed that the protein levels of FAS, FASL, BAX, BCL2, and GSK3β in the control cells (normoxia) reached 0.06, 134.43, 19.07, 1.47, and 2.17 pg per mg of total protein, respectively. Hypoxia increased the expression of all analyzed proteins by 46–140%, and PaPE-1 (5 μM) treatment partially inhibited the excessive expression of FAS (108% decrease), FASL (60% decrease), BAX (42% decrease), and GSK3β (71% decrease); however, the expression of BCL2 increased by 360% in response to PaPE-1 treatment. Similar to those under hypoxia, the protein levels of FAS, FASL, BAX, BCL2, and GSK3β significantly increased by 44–262% in response to ischemic conditions. Exposure to PaPE-1 (5 μM) decreased the protein expression of FAS (102% decrease), FASL (50% decrease), BAX (65% decrease), and GSK3β (127% decrease), whereas the expression of BCL2 increased by 111% in response to PaPE-1 treatment. Normoxic cells treated with PaPE-1 did not exhibit changes in the expression of apoptosis-related proteins (Fig. 7b).

## Discussion

Newly synthesized PaPE-1 selectively activates membrane (nonnuclear/extranuclear) estrogen receptors (ERs), namely, mERα, and mERβ, and has been shown to evoke neuroprotection in a cellular model of AD and in a mouse model of stroke, specifically transient middle cerebral artery occlusion (tMCAO). PaPE-1 appeared to inhibit both Aβ-induced neurotoxicity in mouse primary neurons in in vitro cultures and tMCAO-evoked infarction and leukocyte infiltration into the ischemic mouse brain (Wnuk et al. 2020a; Selvaraj et al. 2018). However, until recently, PaPE-1’s effectiveness in protecting brain tissue against hypoxia/ischemia has been verified only in a pretreatment paradigm, namely, PaPE-1 was administered 24 h before inducing brain lesions *via* tMCAO.

This is the first study showing that 6-h delayed posttreatment with PaPE-1 inhibited hypoxia-/ischemia-induced neuronal death, as indicated by neutral red uptake in mouse primary neocortical cell cultures in vitro. The effect was accompanied by substantial decreases in neurotoxicity and neurodegeneration measured by LDH release and Fluoro-Jade C staining of damaged cells, respectively. Previously, we used the same cellular models of perinatal asphyxia and stroke, i.e., 6-h hypoxia or ischemia followed by 18-h reoxygenation, which reflected the major features of brain pathologies in vivo (Wnuk, Przepiórska et al. 2021), including oxidative stress due to enhanced ROS production, oxidative DNA damage, neurotoxicity, and neurodegeneration caused by elevated LDH release and Fluoro-Jade C staining, and neuronal cell death due to decreased MTT activity.

Since PaPE-1 has only been recently synthesized, no relevant reports are available to compare the results of our present studies. The beneficial effects of ER agonists, particularly estrogens, on brain tissue undergoing hypoxia and/or ischemia have been extensively reported; however, only a few research reports have focused on membrane ERs, mainly GPER1 (previously GPR30). In the mouse brain challenged with MCAO-induced ischemic injury, 17β-estradiol (E2)-activated GPR30 was shown to inhibit Toll-like receptor 4 (TLR4)-mediated microglial inflammation as well as infarction volume and neuronal damage (Zhang et al. 2018). Similarly, GPER1 appeared to mediate E2-exerted neuroprotection in mouse hippocampal neurons exposed to oxygen and glucose deprivation (OGD) (Zhao et al. 2016). Activation of GPER1 by the selective agonist G1 exerted anti-inflammatory effects and preserved cognitive function in rats subjected to global cerebral ischemia (Bai et al. 2020). Intriguingly, in female mice subjected to cerebral ischemia, G1 appeared to reduce infarct volume and neurological deficits, whereas in male mice, G1 increased infarct volume and worsened functional outcome (Broughton et al. 2014).

Much less is known about the involvement of non-GPER membrane ERs in neuroprotection against hypoxic/ischemic injury. GPER1 mainly involves PKA, PKB, and ERK signaling cascades and phosphoinositide-3-kinase (PI3K), in addition to G-proteins (Gα_s_, Gα_i_); in contrast, non-GPER membrane ERs act preferably *via* Ca^2+^ liberation from intracellular stores, PLC, PKC, Src/ERK, PI3K/AKT, p38/MAPK, JAK/STAT, Pak1, and FAK (Acconcia et al. 2021). A recently identified membrane-based estrogen receptor, ER-α36, that is expressed in human and rodent brains exhibited neuroprotective properties in animal and cellular models of ischemic stroke (Zou et al. 2015). In particular, ER-α36 was activated by E2 in OGD-exposed PC12 cells, which confirmed its participation in rescuing the cells from ischemic damage. In rat brains subjected to global hypoxia, *Cicer microphyllum* seed supplementation caused neuroprotection that was mediated through ERβ-dependent extranuclear activation of ERK1/2 (Sharma et al. 2017), which is partially in line with the mERα/mERβ-mediated neuroprotection observed in our present study after treatment with PaPE-1.

In this study, the mechanisms of neuroprotective action of PaPE-1 against hypoxia-/ischemia-induced injury also involved an inhibition of apoptosis that was evidenced by normalization of both mitochondrial membrane potential and expression levels of apoptosis-related genes, i.e., *Fas*, *Fasl*, and *Bcl2*, and proteins such as FAS, FASL, BCL2, BAX, and GSK3β. Furthermore, in the present study, PaPE-1-evoked neuroprotection was mediated through a reduction in ROS formation and enhancement of cellular metabolic activity that was dysregulated in the course of hypoxia and ischemia. Previously, we showed that PaPE-1 has the ability to reduce ROS formation and to prevent apoptosis through the restoration of the mitochondrial membrane potential and BAX/BCL2 ratio as well as the downregulation of *Fas*/FAS expression (Wnuk et al. 2020a), which concurs with the results of the present research. Interestingly, estrogen deficiency appeared to predispose brain neurons to apoptosis following cerebral ischemia (Guo et al. 2017), which supports essential roles of estrogen-based compounds such as PaPE-1 in pharmacotherapy of stroke and perinatal asphyxia.

## Conclusion

These data provide evidence that targeting membrane non-GPER estrogen receptors with PaPE-1 is an effective therapy that protects brain neurons from hypoxic/ischemic damage by inhibiting neurotoxicity, neurodegeneration, and apoptosis and by reducing oxidative stress and enhancing cellular metabolic activity, even when applied with a 6-h delay from injury onset. Hence, investigating the molecular mechanisms by which PaPE-1 regulates hypoxia-/ischemia-related processes may lead to the development of a novel posttreatment therapy that targets the membrane-associated non-GPER ERs and opens up new therapeutic perspectives for stroke and perinatal asphyxia.

## Abbreviations

AD: Alzheimer’s disease
Aβ: Amyloid β
E2: 17β-Estradiol
ERs: Estrogen receptors
ERα/β: Estrogen receptor α/β
FJ-C: Fluoro-Jade C
LDH: Lactate dehydrogenase
MCAO: Middle cerebral artery occlusion
mERs: Membrane estrogen receptors
MTT: 3-(4,5-Dimethylthiazol-2-yl)-2,5-diphenyltetrazolium bromide
OGD: Oxygen and glucose deprivation
PaPE-1: Pathway preferential estrogen-1
ROS: Reactive oxygen species
rtPA: Recombinant tissue plasminogen activator
tMCAO: Transient middle cerebral artery occlusion
